# Higher systemic immune-inflammation index is associated with increased risk of erectile dysfunction: Result from NHANES 2001–2004

**DOI:** 10.1097/MD.0000000000035724

**Published:** 2023-11-10

**Authors:** Lian Zhong, Xiangpeng Zhan, Xin Luo

**Affiliations:** a Department of Blood Transfusion, Pingxiang People’s Hospital, Pingxiang, Jiangxi, China; b Department of Dermatology, The Second Xiangya Hospital of Central South University, Hunan Key Laboratory of Medical Epigenomics, Changsha, China.

**Keywords:** association, erectile dysfunction, NHANES, prognostic factors, systemic immune-inflammation index

## Abstract

This study utilized data from the National Health and Nutrition Examination Survey (NHANES) to investigate the association between the systemic immune-inflammation index (SII) and erectile dysfunction (ED) in adult males. The SII is a novel index derived from the counts of neutrophils, lymphocytes, and platelets in the peripheral blood and serves as a comprehensive indicator of the immune response and inflammation levels. The study included 3601 participants from the NHANES 2001-2004 cycle. Covariates such as age, race, marital status, education, smoking, alcohol consumption, BMI, hypertension, and diabetes were taken into account. Weighted analysis and logistic regression models were applied to assess the relationship between SII and ED, adjusting for potential confounding factors. The prevalence of ED was found to be 6.28%. Overall, there is a linear correlation between SII (nonlinear *P* > .05) and ED. After adjusting for various confounding factors, a significant association was observed between high levels of the SII and ED. The odds ratio (OR) for ED in individuals with high SII levels was 1.45 (95% CI: 1.01–2.17, *P* = .045). Subgroup analysis further identified specific participant subgroups with a significant association between SII and ED. Our findings suggest that higher levels of the SII are independently associated with an increased risk of ED in adult males. The SII may serve as a valuable biomarker for identifying individuals at higher risk of ED and may aid in the development of tailored treatment approaches. Further research is needed to explore the underlying mechanisms and potential therapeutic implications.

## 1. Introduction

Erectile dysfunction (ED), a condition characterized by the inability to achieve or maintain an erection sufficient for satisfactory sexual performance, was considered a prevalent ailment among elderly men. Two landmark studies, the Massachusetts Male Aging Study and the European Male Aging Study, have provided valuable insights in this regard.^[1,2]^ The MMAS revealed a comprehensive prevalence rate of 52% for mild to moderate erectile dysfunction among men aged 40 to 70. The occurrence of erectile dysfunction was closely associated with age, overall health status, and emotional well-being.^[1]^ In contrast, the EMAS, the largest multicenter population study focusing on elderly individuals aged 40 to 79, reported prevalence rates ranging from 6% to 64%, depending on different age subgroups. The prevalence increased with age, averaging at 30%.^[2]^ Few studies have evaluated the global prevalence of erectile dysfunction.^[3,4]^ Conclusions drawn from these studies suggest that the systematic prevalence of erectile dysfunction is higher in the United States, as well as in East Asian and Southeast Asian countries, compared to Europe or South America. Several factors may account for these differences, including cultural or socio-economic variables.

In 2014, Hu et al proposed the systemic immune-inflammation index (SII) as a measure to assess local immune responses and systemic inflammation in the human body.^[5]^ It has been observed through various studies that SII can effectively reflect the balance between inflammatory reactions and immunological responses in patients with malignant tumors.^[6,7]^ As a result, SII is currently being utilized as a prognostic indicator in carcinoma research.^[8,9]^ However, there is a scarcity of research on the impact of SII on erectile dysfunction. While SII has been extensively studied in relation to cancer, its association with erectile dysfunction remains largely unexplored. Further investigation is needed to determine the possible connection between SII and erectile dysfunction, as this could potentially shed light on novel therapeutic approaches and prognostic indicators for this condition.

Therefore, our preliminary objective of the study is to evaluate the relationship between SII and ED using data from the National Health and Nutrition Examination Survey (NHANES). Our study is the first large-scale cross-sectional investigation assessing the association between SII and ED, offering new insights into ED management from a new and convenient indicator.

## 2. Materials and Methods

### 2.1. Data and sample sources

The study utilized data from the NHANES, a comprehensive survey conducted by the National Center for Health Statistics. NHANES aims to collect representative information on the health and nutrition of the non-institutionalized civilian population in the United States. In order to ensure a diverse sample, NHANES adopts a stratified, multistage probability approach to select participants from across the country. The survey gathers data through standardized in-home interviews, physical examinations, and laboratory tests carried out at mobile examination centers.

**Figure 1.**
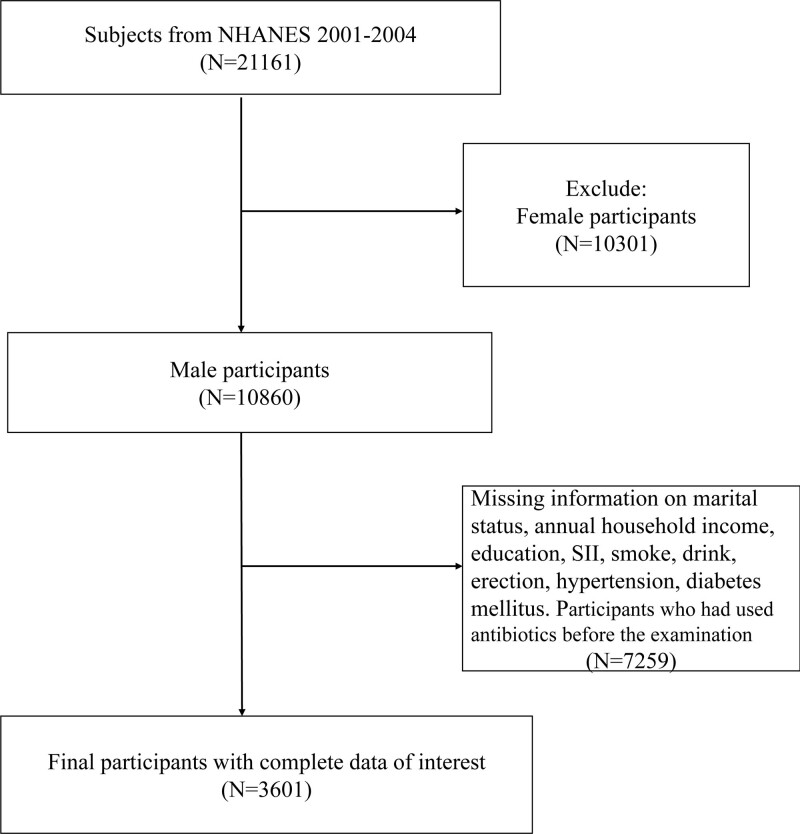
Overview of participants screening. ED = erectile dysfunction, NHANES = National Health and Nutrition Examination Survey, SII = immune-inflammation index.

In this specific study, our focus was on adult males from the NHANES 2001-2004 cycle. The initial sample comprised 21,161 participants. To ensure the reliability of the data, we excluded individuals with missing information on certain variables, including SII, ED, marital status, annual household income, education, smoke and drink, hypertension, and diabetes. In addition, participants who had used antibiotics before the examination were also excluded. This resulted in the inclusion of 3601 participants in the final analysis (Fig. 1).

### 2.2. Assessment systemic immune-inflammation index

The systemic immune-inflammation index (SII) was first introduced by Hu et al^[5]^ as a useful tool for evaluating the prognostic significance of various diseases. This innovative index is derived from the counts of neutrophils (N), lymphocytes (L), and platelets (P) in the peripheral blood. Its calculation involves multiplying the platelet count by the ratio of neutrophil to lymphocyte counts (P × N/L, in 10^9/L). By utilizing the SII, researchers were able to investigate the association between this index and disease outcomes, exploring it both as a continuous variable and as a categorical variable categorized by quartiles.

The SII provides valuable insights into the immune and inflammatory responses within the body. By incorporating multiple components of the immune system, including neutrophils, lymphocytes, and platelets, the index offers a comprehensive perspective on the systemic immune response and inflammation levels. It serves as a precise and quantitative indicator of the delicate interplay between these components.

In their study, Hu et al demonstrated the potential of the SII as a prognostic biomarker for various diseases. By analyzing its association with disease outcomes, they were able to establish a clear relationship between the SII and prognosis. This information aids in the identification of patients who may be at higher risk or require tailored treatment approaches.

### 2.3. Erectile dysfunction assessment

The definition of erectile dysfunction is based on the responses of participants to a survey question: “How would you describe your ability to get and keep an erection adequate for satisfactory intercourse?” If the participants answer “Never able,” it is recorded as erectile dysfunction, while responses indicating “Always or almost always able,” “Sometimes able,” and “Usually able” are recorded as non-erectile dysfunction.

### 2.4. Covariates

The study incorporated several covariates that could potentially affect the association between SII and ED. These covariates encompassed various demographic characteristics of the population such as age, sex, ethnicity, marital status, educational level, smoking status, alcohol consumption, BMI (body mass index), and annual household income. Additionally, health risk factors including diabetes and hypertension were taken into account. More comprehensive categorial data can be found in Table 1.

**Table 1:** Characteristics of the study population by ED.

Variable	Total	Non-ED	ED	*P* value
	3601 (100%)	3185 (100%)	416 (100%)	
Age (mean, SE, year)	45.06 (0.37)	43.52 (0.33)	68.02 (0.81)	<.0001*
SII (mean, SE)	575.74 (6.68)	571.26 (6.9)	642.52 (27.93)	.0214*
<1000	92.14%	92.38%	88.55%	.016*
>1000	7.86%	7.62%	11.45%	
Race				
Non-hispanic black	9.19%	9.41%	6.00%	.0195
Non-hispanic white	74.77%	74.30%	81.89%	
Other	16.03%	16.30%	12.12%	
Marital status				
Married	63.95%	63.12%	76.40%	<.0001*
SDW	11.62%	11.20%	17.89%	
Unmarried	24.42%	25.68%	5.72%	
Annual household income				
0–19.999	13.81%	13.25%	22.15%	<.0001*
20.000–54.999	40.02%	39.15%	52.98%	
55.000–74.999	15.33%	15.62%	11.01%	
75.000	30.85%	31.98%	13.85%	
Education				
High school graduate or under	43.77%	42.88%	57.11%	<.0001*
Some college or above	56.23%	57.12%	42.89%	
Smoke				
Former	29.16%	27.13%	59.34%	<.0001*
Never	42.52%	43.47%	28.41%	
Now	28.32%	29.40%	12.25%	
Alcohol consumption				
No	23.68%	22.15%	46.55%	<.0001*
Yes	76.32%	77.85%	53.45%	
Diabetes				
DM	10.07%	8.60%	31.94%	<.0001*
IFG	3.85%	3.54%	8.52%	
No	86.08%	87.86%	59.55%	
Hypertension				
No	65.29%	67.43%	33.32%	<.0001*
Yes	34.71%	32.57%	66.68%	
BMI (kg/m^2^)				
<25	29.64%	29.94%	25.11%	.0607
25–29.9	41.02%	41.21%	38.20%	
≥30	29.35%	28.85%	36.69%	

BMI = body mass index, DM = diabetes, ED = erectile dysfunction, IFG = impaired fasting glucose, SDW = Separated, Divorced, Widowed, SE = standard error, SII = systemic immune inflammation index.

* Statistical significance.

### 2.5. Statistical analyses

Weighted analysis was performed in accordance with NHANES recommendations to address the complex sampling design in the study. To compare baseline characteristics between the normal group and the ED group, weighted Student t tests were used for continuous variables, while weighted chi-square tests were used for categorical variables. Multivariable logistic regression analysis was conducted to assess the correlation between SII and ED, employing different models.

Model 1 was unadjusted for confounding variables, while Model 2 was adjusted for age and race. Model 3 further took into account factors such as, marital status, annual household income, education level, smoke and drink, BMI, hypertension, and diabetes. Additionally, subgroup analysis based on age, race, marital status smoke and drink was performed to explore the relationship between SII and different ED subgroups.

Data extraction and analysis were carried out using the “nhanesR” package. A significance level of *P* < .05 (two-sided) was set to determine statistically significant differences.

## 3. Results

The prevalence of ED was 6.28%. ED participants are characterized by being older, having a higher proportion of non-Hispanic white individuals, more married participants, lower educational attainment, more former smokers, fewer alcohol drinkers, and a higher prevalence of diabetes and hypertension compared to the general population (all *P* < .05). The SII of ED participants was slightly higher than normal participants (Table 1). Individuals with higher levels of SII exhibit characteristics such as older age, lower economic status, higher smoking rates, a higher proportion of individuals with hypertension, and lower BMI (all *P* < .05, Table 2).

**Table 2:** Characteristics of the study population by SII.

Variable	Total	SII < 1000	SIIS ≥ 1000	*P* value
Age (mean, SE, year)	45.06 (0.38)	44.86 (0.39)	47.47 (1.00)	.02
Erection				
No	93.72%	93.96%	90.85%	.02
Yes	6.28%	6.04%	9.15%	
Race				
Non-hispanic black	9.19%	9.49%	5.64%	.09
Non-hispanic white	74.77%	74.39%	79.23%	
Other	16.03%	16.11%	15.13%	
Marital status				
Married	63.95%	64.66%	55.73%	.04
SDW	11.62%	11.32%	15.16%	
Unmarried	24.42%	24.02%	29.11%	
Annual household income				
0–19.999	13.81%	13.60%	16.25%	.01
20.000–54.999	40.02%	39.38%	47.48%	
55.000–74.999	15.33%	15.33%	15.27%	
75.000	30.85%	31.69%	21.00%	
Education				
High school graduate or under	43.77%	43.36%	48.61%	.19
Some college or above	56.23%	56.64%	51.39%	
Smoke				
Former	29.16%	29.09%	29.91%	.04
Never	42.52%	43.19%	34.67%	
Now	28.32%	27.71%	35.42%	
Alcohol consumption				
No	23.68%	23.92%	20.88%	.34
Yes	76.32%	76.08%	79.12%	
Diabetes				
DM	10.07%	10.10%	9.66%	.89
IFG	3.85%	3.83%	4.08%	
No	86.08%	86.07%	86.26%	
Hypertension				
No	65.29%	65.75%	59.81%	.02
Yes	34.71%	34.25%	40.19%	
BMI (kg/m^2^)				
<25	29.64%	28.90%	38.27%	.01*
25–29.9	41.02%	41.19%	39.03%	
≥30	29.35%	29.91%	22.69%	

BMI = body mass index, DM = diabetes, ED = erectile dysfunction, IFG = impaired fasting glucose, SDW = Separated, Divorced, Widowed, SE = standard error, SII = systemic immune inflammation index.

* Statistical significance.

After adjusting for other potential confounding factors, we established multiple models to evaluate the independent influence of SII on ED. In the univariate logic analysis, we observed a significant association (*P* < .05, Table S1, Supplemental Digital Content, http://links.lww.com/MD/K582) between SII and a higher incidence of ED, along with factors such as age, diabetes, race, education, marital status, household income, diabetes, smoking, alcohol consumption, and hypertension.

**Figure 2.**
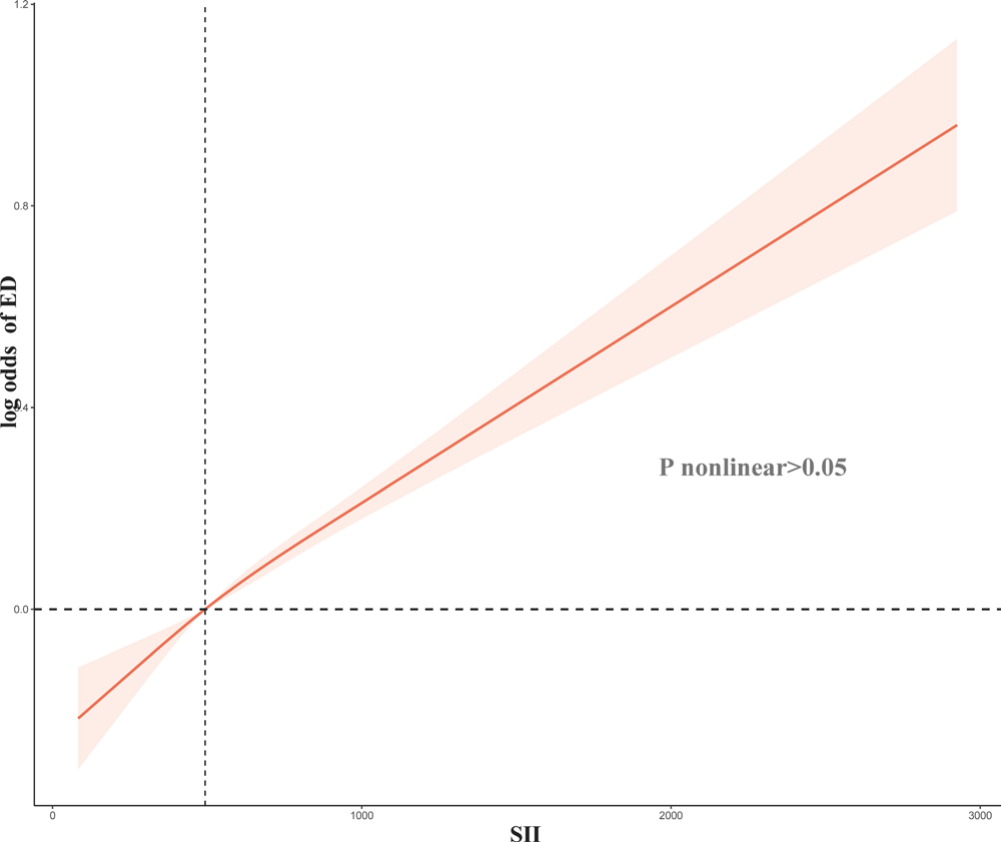
Relation of systemic immune-inflammation index (SII) with risk of erectile dysfunction (ED).

Overall, there is a linear correlation between SII (nonlinear *P* > .05, Fig. 2) and ED. The risk of developing ED increases gradually with an increase in SII. Through analysis using a Logit model, it has been demonstrated that SII levels have an independent and significant association with ED (OR: 1.0004, 95% CI: 1.0002–1.0006, *P* < .001). Univariate analysis revealed that higher levels of SII pose a risk factor for ED (≥1000 vs <1000, OR = 1.57, 95% CI: 1.09–2.25, *P* = .02, Table 3). After adjusting for age and race, the model still indicates a significant correlation between high level of SII levels and a higher incidence of ED (OR: 1.50, 95% CI: 1.05–2.14, *P* = .03). Finally, after adjusting for age, race, marital status, family income, education, smoking, alcohol consumption, BMI, diabetes, and hypertension, a multivariable logistic regression model demonstrated that high level SII remained associated with ED (OR: 1.45, 95% CI: 1.01–2.17, *P* = .045).

**Table 3:** Multivariate logistic regression models of ED and SII.

DII	Model 1		Model 2		Model 3	
	OR (95% CI)	*P* value	OR (95% CI)	*P* value	OR (95% CI)	*P* value
Continuous	1.0004 (1.0002, 1.0006)	<.001*	1.0003 (1.0001, 1.0006)	.02*	1.0002 (1.0001, 1.001)	.04*
<1000	Ref.		Ref.		Ref.	
≥1000	1.57 (1.09, 2.25)	.02*	1.50 (1.05, 2.14)	.03*	1.45 (1.01, 2.17)	.045*

Model 1: adjust for non.

Model 2: adjust for age, race.

Model 3: adjust for Age, Race, Marital status, Annual household income, Education, Smoke, Drink, BMI, Diabetes, Hypertension.

CI = confidence interval, ED = erectile dysfunction, OR = odd ratio, SII = systemic immune-inflammation index.

* Statistical significance.

When conducting subgroup analysis based on age, race, marital status, education, smoking, alcohol consumption, and BMI, we found a significant association between SII and ED occurrence in the following participant subgroups (Table 2): individuals aged over 50, non-Hispanic white, married individuals, never smoke, participants with a history of alcohol consumption, education of some college or above, and those with BMI of 25 to 29.9 (all *P* < .05) (Table 4).

**Table 4:** Multivariate logistic regression models of SII on ED by age, race, marital status, smoke, drinks, education, BMI.

Character	SII < 1000	SII ≥ 1000	*P* value
Age (yr)			
30–50	ref	1.37 (0.26, 7.14)	.7
>50	ref	1.60 (1.09, 2.34)	.02*
<30	ref	0.54 (0.07, 3.85)	.52
Race			
Non-hispanic white	ref	1.60 (1.04, 2.48)	.03*
Other	ref	1.19 (0.58, 2.41)	.62
Non-hispanic black	ref	1.17 (0.58, 2.38)	.65
Marital status			
SDW	ref	1.68 (0.86, 3.27)	.13
Married	ref	1.75 (1.15, 2.66)	.01*
Unmarried	ref	0.71 (0.15, 3.33)	.65
Drinks			
Yes	ref	1.68 (1.16, 2.45)	.01*
No	ref	1.61 (0.85, 3.05)	.14
Smoke			
Now	ref	0.91 (0.26, 3.19)	.87
Former	ref	1.41 (0.84, 2.36)	.19
Never	ref	2.62 (1.38, 4.98)	.005*
Education			
Some college or above	ref	2.01 (1.16, 3.48)	.01*
High school graduate or under	ref	1.23 (0.78, 1.94)	.37
BMI (kg/m^2^)			
≥30	ref	1.47 (0.85, 2.55)	.16
<25	ref	1.27 (0.60, 2.67)	.52
25–29.9	ref	2.09 (1.17, 3.75)	.01*

BMI = Body Mass Index, ED = Erectile dysfunction, SDW = Separated, Divorced, Widowed, SE = Standard error, SII = Systemic immune inflammation index.

* Statistical significance.

## 4. Discussion

In summary, SII was significantly associated with an increased incidence of ED, even after adjusting for potential confounding factors. Higher levels of SII pose a risk factor for ED, and there is a linear correlation between SII and ED. This association holds true across different age groups, races, marital statuses, education levels, smoking and alcohol habits, and BMI categories.

Li et al^[10]^ had pointed out a correlation between high SII and testosterone deficiency. Testosterone, one of the primary sex hormones in males, played a crucial role in erectile function. Thus, the correlation between SII and male erectile function is understandable. Additionally, some studies have found a link between high SII and depression and hyperlipidemia.^[11,12]^ Depression and hyperlipidemia could, to some extent, affect male erectile function. Therefore, these research findings further support the notion that SII was related to male erectile function. In summary, previous research suggested that SII was associated with testosterone deficiency, depression, and hyperlipidemia, all of which are closely related to male erectile function. Hence, it is reasonable to infer that there is a relationship between SII and male erectile function. These research findings helped us gain a more comprehensive understanding of sexual dysfunction and facilitate further studies in related fields.

Inflammation was widely recognized as a significant contributor to the onset of ED. Numerous studies indicate that individuals with ED, as well as rats involved in research, exhibit heightened levels of inflammatory factors such as interleukin (IL)-6, IL-1β, and TNF-α.14.^[13]^ Additionally, Araña Rosaínz et al^[14]^ found that diabetic patients with a reduced risk of ED often displayed elevated levels of the anti-inflammatory factor IL-10. Such evidence strongly suggests that systemic inflammation plays a crucial role in the development of ED. It is worth noting that various inflammatory molecules have shown promise in assessing the risk and treatment efficacy of ED. Several studies have linked the severity of ED, as well as its presence, to markers and mediators of subclinical inflammation and endothelial dysfunction.^[15]^ Furthermore, research has revealed a negative association between 5-item International Index of Erectile Function scores and the levels of fibrinogen, IL-1β, vascular hemophilia factor, and IL-6.^[16]^ While the precise mechanism by which inflammation causes ED remains unclear, there is an undeniable and robust connection between the 2. In an environment predominantly characterized by proinflammatory responses, nitric oxide bioavailability is compromised, resulting in impaired vasodilation. This impairment can be attributed to the suppression of endothelial nitric oxide synthase expression and the overproduction of reactive oxygen species, ultimately leading to endothelial dysfunction^[17]^ Understanding the intricate interplay between inflammation and ED is crucial for advancing effective treatment strategies and interventions.

The SII was determined based on the counts of 3 types of circulating immune cells: neutrophils, lymphocytes, and platelets. The level of SII reflected the inflammatory state and could serve as an easily detectable biomarker for systemic inflammation activity.^[18]^ Our research findings demonstrated that individuals with ED had significantly higher levels of SII compared to those without ED, and elevated SII levels are an independent risk factor for ED. After controlling for potential confounding factors, we found a correlation between SII and ED. Patients with high SII levels commonly suffer from thrombocytosis, neutrophilia, or lymphocytopenia.^[19]^ Lymphocytes and neutrophils mediate adaptive and innate immunity, respectively. Neutrophils, being the largest proportion of white blood cells, play a crucial role in initiating and regulating immune processes and secrete neutrophil elastase to mediate chronic inflammation.^[6]^ Patients with increased neutrophil activity release reactive oxygen species, which may be associated with the development of ED. Lymphocytes, on the other hand, are integral components of white blood cells, participating in adaptive immunity and aiding in innate immunity. They serve as specific inflammatory mediators with regulatory or protective functions.

Psychological factors might play an important role in the relationship between the SII and ED. Prolonged psychological distress, such as anxiety, depression, stress, and psychological trauma, might directly or indirectly affect the body’s inflammatory response and erectile function. Psychological distress can trigger changes in the immune system, increasing inflammatory response, and long-term inflammation can have negative effects on gonadal function and blood supply. Additionally, psychological distress and inflammation might also contribute to the occurrence of erectile dysfunction. Psychological distress may disrupt the balance between the sympathetic and parasympathetic nervous systems, affecting the physiological processes of erection, while inflammation may negatively impact erectile function through various pathways. These factors form a complex network of interactions, where psychological distress may trigger inflammatory response, and the presence of inflammation may exacerbate psychological distress, perpetuating a cycle of mutual reinforcement. This cycle can lead to long-term physical and psychological discomfort and sexual dysfunction.

When investigating the association between SII and ED, Chunhui Liu et al did not observe any significant relationship in their multivariate logistic regression model.^[17]^ The inconsistent findings could potentially be attributed to the exclusion of a substantial number of study participants during the analysis of various inflammatory indicators. This limitation may have contributed to the lack of association observed between SII and ED. However, it is important to note that our study did identify a significant association between an SII threshold above 1000 and an increased risk of developing ED. Furthermore, we conducted subgroup analyses in multiple populations, which provided additional valuable insights.

Our study aimed to comprehensively investigate the relationship between SII and ED. Previous research had already demonstrated the significant predictive capability of SII, and its widespread availability, non-invasiveness, user-friendly nature, and affordability make it highly promising for clinical applications. There were several strengths in our study. Firstly, we have utilized the largest sample size currently available in this field of research. Secondly, we have meticulously considered potential confounding variables to enhance the reliability and validity of our findings. However, several limitations should be acknowledged. Firstly, due to the cross-sectional design of the NHANES dataset, we were unable to establish causality between SII and ED. Secondly, potential biases might have been introduced during the data collection process, particularly through interviews and online forums. Thirdly, although adjustments had been made for several relevant confounding factors, the influence of other latent confounders might not have been entirely eliminated. Lastly, the immune cell counts incorporated in our analysis were derived from a single blood test. Considering the limited lifespan of blood cells, continuous monitoring might offer more reliable and accurate results compared to a single measurement.

## 5. Conclusion

In conclusion, we found a significant association between high levels of SII and the incidence of ED. This association was observed across different participant subgroups, including age, race, marital status, education level, smoking, alcohol consumption, and BMI. This suggests that SII may serve as an independent predictive factor for ED and play an important role in identifying high-risk patients and developing personalized treatment approaches. However, further research is still needed to validate this association and gain deeper insights into the mechanisms underlying the relationship between SII and ED.

## Author contributions

**Conceptualization:** Zhong Lian, Xiangpeng Zhan, Xin Luo.

**Data curation:** Zhong Lian, Xiangpeng Zhan, Xin Luo.

**Formal analysis:** Xiangpeng Zhan, Xin Luo.

## Supplementary Material



## References

[1] Feldman HA Goldstein I Hatzichristou DG. Impotence and its medical and psychosocial correlates: results of the Massachusetts male aging study. J Urol. 1994;151:54–61. 8254833 10.1016/s0022-5347(17)34871-1

[2] Corona G Lee DM Forti G.; EMAS Study Group. Age-related changes in general and sexual health in middle-aged and older men: results from the European Male Ageing Study (EMAS). J Sex Med. 2010;7(4 Pt 1):1362–80. 19929914 10.1111/j.1743-6109.2009.01601.x

[3] Rosen R Altwein J Boyle P. Lower urinary tract symptoms and male sexual dysfunction: the multinational survey of the aging male (MSAM-7). Eur Urol. 2003;44:637–49. 14644114 10.1016/j.eururo.2003.08.015

[4] Nicolosi A Laumann EO Glasser DB.; Global Study of Sexual Attitudes and Behaviors Investigators' Group. Sexual behavior and sexual dysfunctions after age 40: the global study of sexual attitudes and behaviors. Urology. 2004;64:991–7. 15533492 10.1016/j.urology.2004.06.055

[5] Hu B Yang XR Xu Y. Systemic immune-inflammation index predicts prognosis of patients after curative resection for hepatocellular carcinoma. Clin Cancer Res. 2014;20:6212–22. 25271081 10.1158/1078-0432.CCR-14-0442

[6] Chen JH Zhai ET Yuan YJ. Systemic immune-inflammation index for predicting prognosis of colorectal cancer. World J Gastroenterol. 2017;23:6261–72. 28974892 10.3748/wjg.v23.i34.6261 PMC5603492

[7] Miao Y Yan Q Li S. Neutrophil to lymphocyte ratio and platelet to lymphocyte ratio are predictive of chemotherapeutic response and prognosis in epithelial ovarian cancer patients treated with platinum-based chemotherapy. Cancer Biomark. 2016;17:33–40. 27314290 10.3233/CBM-160614 PMC13020469

[8] Ren A Li Z Cheng P. Systemic immune-inflammation index is a prognostic predictor in patients with intrahepatic cholangiocarcinoma undergoing liver transplantation. Mediators Inflamm. 2021;2021:6656996. 33628115 10.1155/2021/6656996 PMC7899762

[9] Fu H Zheng J Cai J. Systemic immune-inflammation index (sii) is useful to predict survival outcomes in patients after liver transplantation for hepatocellular carcinoma within Hangzhou criteria. Cell Physiol Biochem. 2018;47:293–301. 29768257 10.1159/000489807

[10] Li Y Liu M Cui Y. Increased risk of testosterone deficiency is associated with the systemic immune-inflammation index: a population-based cohort study. Front Endocrinol. 2022;13:974773. 10.3389/fendo.2022.974773 PMC9424499 36051392

[11] Wang J Zhou D Dai Z. Association between systemic immune-inflammation index and diabetic depression. Clin Interv Aging. 2021;16:97–105. 33469277 10.2147/CIA.S285000 PMC7810592

[12] Mahemuti N Jing X Zhang N. Association between systemic immunity-inflammation index and hyperlipidemia: a population-based study from the NHANES (2015-2020). Nutrients. 2023;15:1177. 36904176 10.3390/nu15051177 PMC10004774

[13] Hu Y Niu X Wang G. Chronic prostatitis/chronic pelvic pain syndrome impairs erectile function through increased endothelial dysfunction, oxidative stress, apoptosis, and corporal fibrosis in a rat model. Andrology. 2016;4:1209–16. 27565759 10.1111/andr.12273

[14] Araña Rosaínz MJ Ojeda MO Acosta JR. Imbalanced low-grade inflammation and endothelial activation in patients with type 2 diabetes mellitus and erectile dysfunction. J Sex Med. 2011;8:2017–30. 21554550 10.1111/j.1743-6109.2011.02277.x

[15] Corona G Mannucci E Schulman C. Psychobiologic correlates of the metabolic syndrome and associated sexual dysfunction. Eur Urol. 2006;50:595–604; discussion. 16564129 10.1016/j.eururo.2006.02.053

[16] Vlachopoulos C Aznaouridis K Ioakeimidis N. Unfavourable endothelial and inflammatory state in erectile dysfunction patients with or without coronary artery disease. Eur Heart J. 2006;27:2640–8. 17056702 10.1093/eurheartj/ehl341

[17] Liu C Gao Y Ji J. Association between inflammatory indexes and erectile dysfunction in U.S. adults: National Health and Nutrition Examination Survey 2001-2004. Sexual Med. 2023;11:qfad045. 10.1093/sexmed/qfad045 PMC10413424 37577069

[18] Lolli C Caffo O Scarpi E. Systemic immune-inflammation index predicts the clinical outcome in patients with mCRPC treated with abiraterone. Front Pharmacol. 2016;7:376. 27790145 10.3389/fphar.2016.00376 PMC5062111

[19] Geng Y Shao Y Zhu D. Systemic immune-inflammation index predicts prognosis of patients with esophageal squamous cell carcinoma: a propensity score-matched analysis. Sci Rep. 2016;6:39482. 28000729 10.1038/srep39482 PMC5175190

