# Supplementary material for: Higher systemic immune-inflammation index is associated with increased risk of erectile dysfunction: Result from NHANES 2001–2004
Source: Medicine (Baltimore). 2023 Nov 10;102(45):e35724. doi: 10.1097/MD.0000000000035724 (PMC10637557; doi:10.1097/MD.0000000000035724)
Supplement: Supplementary file 1 [file medi-102-e35724-s001.docx]

| **Supplement table 1: Univariate logic analysis on factors associated with erectile dysfunction** | | |
| --- | --- | --- |
| **Character** | **95% CI** | **P-value** |
| Age | 1.11(1.10,1.13) | <0.0001 |
| Race |  |  |
| black | ref | ref |
| other | 1.17(0.69,1.96) | 0.55 |
| white | 1.73(1.20,2.49) | 0.005 |
| SII | 1.0004(1.0002,1.0006) | <0.001 |
| Marital status |  |  |
| Married | ref | ref |
| SDW | 1.32(0.88,1.98) | 0.17 |
| unmarried | 0.18(0.11,0.32) | <0.0001 |
| Annual household income |  |  |
| 0-19.999 | ref | ref |
| 20.000-54.999 | 0.81(0.57,1.16) | 0.23 |
| 55.000-74.999 | 0.42(0.26,0.70) | 0.002 |
| 75.000 | 0.26(0.16,0.42) | <0.0001 |
| Education |  |  |
| High school graduate or under | ref | ref |
| Some college or above | 0.56(0.43,0.73) | <0.001 |
| smoke |  |  |
| former | ref | ref |
| never | 0.30(0.22,0.40) | <0.0001 |
| now | 0.19(0.14,0.26) | <0.0001 |
| Alcohol consumption |  |  |
| No | ref | ref |
| Yes | 0.33(0.25,0.43) | <0.0001 |
| DM |  |  |
| DM | ref | ref |
| IFG | 0.65(0.41,1.03) | 0.06 |
| no | 0.18(0.13,0.25) | <0.0001 |
| Hypertension |  |  |
| no | ref | ref |
| yes | 4.14(3.09,5.55) | <0.0001 |
| BMI((kg/m2) |  |  |
| <25 | ref | ref |
| ≥30 | 1.52(1.00,2.30) | 0.05 |
| 25-29.9 | 1.11(0.83,1.47) | 0.47 |
| DII: dietary inflammatory index; SDW: Separated, Divorced, Widowed; DM: Diabetes; IFG: Impaired fasting glucose, BMI: Body Mass Index | | |
